# Epigenome-wide association study of metabolic syndrome in African-American adults

**DOI:** 10.1186/s13148-018-0483-2

**Published:** 2018-04-10

**Authors:** Tomi Akinyemiju, Anh N. Do, Amit Patki, Stella Aslibekyan, Degui Zhi, Bertha Hidalgo, Hemant K. Tiwari, Devin Absher, Xin Geng, Donna K. Arnett, Marguerite R. Irvin

**Affiliations:** 1 Department of Epidemiology, University of Kentucky, Lexington, KY USA; 2 Department of Epidemiology, University of Alabama at Birmingham, Birmingham, AL USA; 3 Department of Biostatistics, University of Alabama at Birmingham, Birmingham, AL USA; 4 School of Biomedical Informatics, University of Texas Health Science Center at Houston, Houston, TX USA; 5 School of Public Health, University of Texas Health Science Center at Houston, Houston, TX USA; 6 HudsonAlpha Institute for Biotechnology, Huntsville, AL USA; 7 College of Public Health, University of Kentucky, Lexington, KY USA

## Abstract

**Background:**

The high prevalence of obesity among US adults has resulted in significant increases in associated metabolic disorders such as diabetes, dyslipidemia, and high blood pressure. Together, these disorders constitute metabolic syndrome, a clinically defined condition highly prevalent among African-Americans. Identifying epigenetic alterations associated with metabolic syndrome may provide additional information regarding etiology beyond current evidence from genome-wide association studies.

**Methods:**

Data on metabolic syndrome and DNA methylation was assessed on 614 African-Americans from the Hypertension Genetic Epidemiology Network (HyperGEN) study. Metabolic syndrome was defined using the joint harmonized criteria, and DNA methylation was assessed using the Illumina HumanMethylation450K Bead Chip assay on DNA extracted from buffy coat. Linear mixed effects regression models were used to examine the association between CpG methylation at > 450,000 CpG sites and metabolic syndrome adjusted for study covariates. Replication using DNA from a separate sample of 69 African-Americans, as well as meta-analysis combining both cohorts, was conducted.

**Results:**

Two differentially methylated CpG sites in the *IGF2BP1* gene on chromosome 17 (cg06638433; *p* value = 3.10 × 10^− 7^) and the *ABCG1* gene on chromosome 21 (cg06500161; *p* value = 2.60 × 10^− 8^) were identified. Results for the *ABCG1* gene remained statistically significant in the replication dataset and meta-analysis.

**Conclusion:**

Metabolic syndrome was consistently associated with increased methylation in the *ABCG1* gene in the discovery and replication datasets, a gene that encodes a protein in the ATP-binding cassette transporter family and is involved in intra- and extra-cellular signaling and lipid transport.

**Electronic supplementary material:**

The online version of this article (10.1186/s13148-018-0483-2) contains supplementary material, which is available to authorized users.

## Background

The prevalence of obesity among US adults has increased steadily over the past few decades, and currently over two thirds of US adults are either obese or overweight [1]. Excess adipose tissue is linked with chronic inflammation, immune system activation, and oxidative stress, as well as hypertension, dyslipidemia, and insulin resistance. This cluster of inter-related conditions is defined clinically as metabolic syndrome (MetS), and the prevalence of this condition has increased concurrently with obesity rates in the USA [2]. The link between MetS and increased risk and mortality due to many chronic diseases such as cardiovascular disease (CVD), arthritis, chronic kidney disease, schizophrenia, and cancer [3–12] has been reported in published studies. More recently, there has been renewed interest in better understanding the biological mechanisms underlying the pathogenesis of MetS.

Epigenetic studies provide a unique lens for clarifying molecular pathways through which lifestyle and environmental factors such as diet and physical activity coupled with individual genetic background interact to influence health outcomes. Alterations in epigenetic patterns, such as DNA methylation, which are modifiable, provide a potential mechanism through which MetS and other environmental factors may influence gene expression and ultimately increasing disease risk or mortality. Like other chronic diseases, marked racial differences exist in the prevalence of MetS and its components [2], and the biological mechanisms underlying such disparities remain unclear. Studies of DNA methylation alterations provide an opportunity to systematically interrogate the epigenome to identify population-specific variation associated with disease risk and health outcomes.

The goal of the present study is to examine DNA methylation signatures measured across the genome associated with MetS using DNA from African-American adults included in the Hypertension Genetic Epidemiology Network (HyperGEN) study, with replication in an independent cohort of participants in the REasons for Geographic and Racial Differences in Stroke (REGARDS) study. Results will enhance understanding of the pathophysiology of this condition among African-Americans and potentially lead to the development of novel biomarkers or molecular targets for clinical studies.

## Methods

### Study population and baseline variables

The Hypertension Genetic Epidemiology Network (HyperGEN) study is a cross-sectional, population-based study of 1224 hypertensive African-American sibships initially recruited between 1996 and 1999. The study was later extended to other siblings and offspring of the original sib-pair. Using stored samples, an ancillary epigenetic study was conducted among cohort participants belonging to the highest and lowest quartiles (total *N* = 636) of left ventricular mass (LV mass). The family structure of this HyperGEN epigenetic study population, including the number of individuals per family, parent-child, and sibling pairs, is presented in Additional file 1: Table S1. Detailed lifestyle, biometric, and laboratory measures were obtained in HyperGEN, including blood pressure, medications, fasting blood glucose, triglycerides, HDL cholesterol, height, weight, and waist circumference. BMI was calculated using standard measures.

### Metabolic syndrome

This was defined using the recently published joint harmonized criteria [13]. The criteria defined MetS as having three or more of the following components: (1) elevated waist circumference (≥ 88 cm for women and ≥ 102 cm for men), (2) elevated triglycerides (≥ 150 mg/dL) or on treatment for dyslipidemia (statin and/or fibric acid derivative), (3) reduced high-density lipoprotein (HDL) cholesterol (< 40 mg/dL in men and < 50 mg/dL in women) or on treatment for dyslipidemia (statin and/or fibric acid derivative), (4) elevated blood pressure (systolic ≥ 130 and/or diastolic ≥ 85 mmHg) or antihypertensive drug treatment in a patient with a history of hypertension), and (5) elevated fasting glucose (≥ 100 mg/dL) or drug treatment for elevated glucose. Of the 636 adults evaluated from the HyperGen cohort, 614 had methylation and MetS data for the basic model; an additional 8 had missing covariate data, leaving 606 for the fully adjusted model.

### Epigenomic analysis

The Illumina HumanMethylation450 array was used to analyze DNA extracted from buffy coat. These arrays can measure DNA methylation at > 480,000 cytosine-phosphate-guanine (CpG) sites. Briefly, 500 ng of buffy coat DNA was hybridized to the Methyl450 array after bisulfite conversion with EZ DNA kits (Zymo Research, Irvine, CA) prior to standard Illumina amplification, hybridization, and imaging steps. The resulting intensity files were analyzed with Illumina’s GenomeStudio, which generated beta (*β*) scores (i.e., the proportion of total signal from the methylation-specific probe or color channel) and “detection *p* values” (probability that the total intensity for a given probe falls within the background signal intensity). Quality control (QC) measures were conducted by removing samples having more than 1% of CpG sites with a detection *p* value > 0.05, removing CpG sites having more than 5% of samples with a detection *p* value > 0.01, and CpG sites with detection *p* value > 0.01 set as missing. After these QC filters, 484,366 CpG sites were eligible for analysis. We normalized the *β* scores using the Subset-quantile Within Array Normalization (SWAN) method in *minifi* package to correct for differences between batches and the type I and type II assay designs within a single 450K array [14]. Cell count proportions (CD8 T lymphocytes, CD4 T lymphocytes, natural killer cells, B cells, and monocytes) were created using the algorithm developed by Houseman, which predicts underlying cellular composition of each sample from DNA methylation patterns [15].

### Available SNP data

GWAS data from HyperGEN has been previously described [16]. African-Americans were genotyped with the Affymetrix Genome Wide Human SNP Array 6.0 Array for 872,242 SNPs (median call rate = 99.97%). This SNP set was used for imputation with ~ 3.01 million HapMap SNPs (release 22, phasing data, “revised union” of CEU and YRI populations) using MACH [17, 18]. The GWAS data described was used to investigate *cis*-methylation quantitative trait loci (*cis*-meQTL) in the region of the statistically significant CpG association findings. HyperGEN-imputed GWAS data was also used to create principal components for ancestry included in epigenetic association models to adjust for subpopulation structure [19].

### Statistical analysis

We determined the proportion of study participants meeting the criteria for MetS and used chi-square tests and *t* tests to examine differences in baseline socio-demographic variables. We fit linear mixed effects regression models with the CpG *β* score (approximating % DNA methylation at a CpG site) as the outcome, MetS as the predictor, and adjusted for age, sex, center, first four principal components (PCs) for ancestry (ancestry PCs), estimated cell proportions, batch effects (array, row, and column to account for residual batch effects after normalization), and family structure as a random effect (R *kinship* package (*lmekin* function)) [20]. In the fully adjusted model, we additionally adjusted for smoking and alcohol use. A sensitivity model for our statistically significant findings was further adjusted for high versus low left ventricular mass. We corrected for multiple comparisons using a Bonferroni correction for 0.05/460,000 tests setting alpha to 1.1 × 10^− 7^. Given that Bonferroni correction tends to be conservative, we also used FDR correction for multiple testing. We constructed Manhattan plots to present results of our epigenome-wide association analysis of MetS and used mixed models to compare methylation of the top CpG sites by MetS and component status adjusting for family structure (R *kinship* package (*lmekin* function)) [20]. To evaluate the DNA sequence contributions to methylation at the top CpG loci, we conducted *cis*-meQTL analyses by interrogating associations between all SNPs within 20 kb of the CpG site via linear mixed models with the CpG *β* score as the outcome, adjusted for age, sex, study site, ancestry PCs, blood cell counts, and familial relationship. For the *cis*-meQTL analysis, we used the Bonferroni correction for multiple testing as 0.05/the number of SNPs within a 20-kb window. Subsequently, significant SNPs from *cis*-meQTL analysis were tested for association with MetS via mixed linear models with MetS as the outcome, adjusted for age, sex, study site, ancestry PCs, and familial relationship. We also conducted lookups of the significant MetS SNPs in the catalog of genome-wide association studies (www.gwascentral.org) to evaluate evidence for association with cardiometabolic traits in other populations.

### Replication analysis

To assess whether similar findings are replicated in a separate study population, we analyzed DNA methylation data obtained from genomic DNA samples of 100 African-American participants in the REasons for Geographic and Racial Differences in Stroke (REGARDS) study also assayed on the Illumina HumanMethylation450 array. In the REGARDS study, blood pressure, fasting blood glucose, triglycerides, HDL cholesterol, and waist circumference were obtained by trained health professionals during in-home visits at study baseline. Details of the REGARDS study have been published previously [21]. In brief, REGARDS is a nationally representative sample of 32,239 adults ages 45 years and older (45% males and 41% African-American) recruited between 2003 and 2007 and followed for incident stroke and other related cardiovascular outcomes. MetS was defined in REGARDS following the joint harmonized criteria, and epigenomic analysis was conducted using data from 69 participants with complete information for both methylation and MetS. Linear models were used to test the association between CpG site methylation and MetS, adjusting for age, sex, four principal components for ancestry, and cell count proportions in the basic model and adding alcohol and smoking in a fully adjusted model. Statistically significant CpGs from the HyperGEN discovery analyses were tested for replication in REGARDS, and a Bonferroni correction was applied (of 0.05/2 significant CpGs = 2.5 × 10^− 2^).

### Meta-analysis

Top results from the HyperGEN and REGARDS studies were meta-analyzed using random effects models implemented in the METASOFT program based on the method of Han and Eskin [22] to ensure validity in the presence of effect heterogeneity.

## Results

Among 614 African-American adults in the HyperGen study (67% female), about half (51%) met the criteria for MetS (Table 1). MetS+ participants were older on average compared with MetS− participants, 52 ± 10 years and 46 ± 11 years, respectively. Body weight (98.5 vs. 84.3 kg), waist circumference (112.4 vs. 96.6 cm), and BMI (35.4 vs. 29.8) were significantly higher in MetS+ compared with MetS− participants. In addition, MetS+ participants had higher levels of triglycerides (125.5 vs. 77 mg/dL) and LDL cholesterol (128 vs. 118.5 mg/dL), lower levels of HDL cholesterol (46.9 vs. 58.8 mg/dL), and higher prevalence of hypertension (87 vs. 49%) compared with MetS− participants. Among MetS+ participants, 51% had three of the five components, 28% had four, and 20% had five, and among MetS− participants, 17% had none of the MetS components, 34% had one, and 49% had two.

**Table 1:** Baseline characteristics of HyperGen study participants

	MetS+ (*N =* 284)	MetS− (*N =* 330)	*p* value*
Age^†^	52.0 ± 10.0	46.0 ± 11.0	< 0.0001
High WC/obesity			
WC (cm)^†^	112.4 ± 16.6	96.6 ± 16.9	< 0.0001
BMI^†^	35.4 ± 8.1	29.8 ± 7.4	< 0.0001
Elevated triglycerides			
Triglycerides (mg/dL)^§^	125.5 ± 83.5	77.0 ± 42.8	< 0.0001
Reduced HDL cholesterol			
HDL cholesterol (mg/dL)^†^	46.9 ± 12.5	58.8 ± 15.5	< 0.0001
Elevated blood pressure			
DBP (mmHg)^†^	75.3 ± 11.8	75.5 ± 13.3	0.8
SBP (mmHg)^†^	136 ± 22.8	127.6 ± 23.7	< 0.0001
Hypertension (%)	86.6	48.9	< 0.0001
Elevated fasting glucose			
Fasting glucose (mg/dL)^†^	133.8 ± 69.9	94.2 ± 31.2	< 0.0001
Metabolic components** (%)			
0	0	17.3	
1	0	33.9	
2	0	48.8	
3	51.4	0	
4	28.2	0	
5	20.4	0	

*BMI* body mass index, *HDL* high-density lipoprotein, *LDL* low-density lipoprotein, *DBP* diastolic blood pressure, *SBP* systolic blood pressure

^†^Presented as mean (standard deviation) for normal continuous characteristics

^§^Presented as median (interquartile range) for non-parametric continuous characteristics

*Significance determined using chi-square test for categorical, *t* test for continuous, or kruskal.test test for non-parametric continuous variables

**Metabolic components are high waist circumference (WC), elevated triglycerides, reduced HDL cholesterol, elevated blood pressure, and elevated fasting glucose

**Fig. 1:**
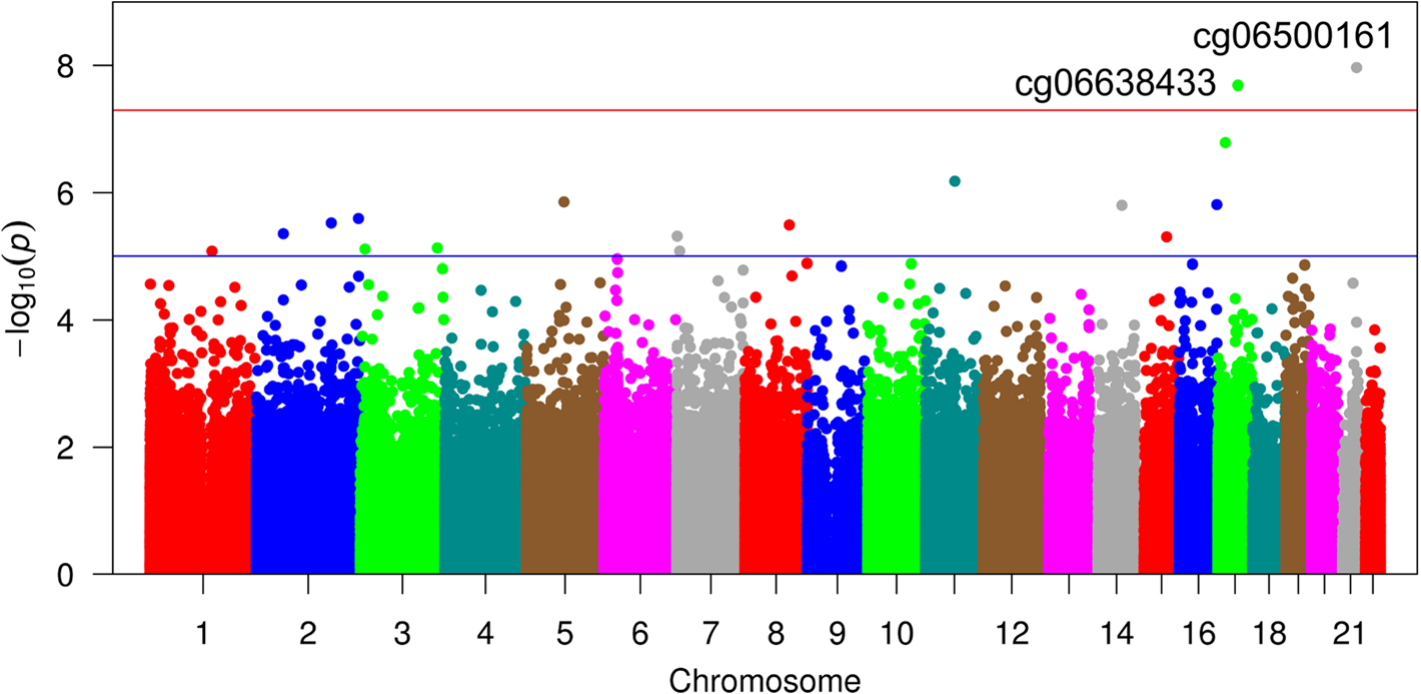
Epigenome-wide association for metabolic syndrome among African-Americans in HyperGEN study (*N* = 614). Basic model includes age, sex, center, first four principal components, five estimated cell proportions, batch effect, and family structure

Two differentially methylated CpG sites—one in the *IGF2BP1* gene on chromosome 17 (cg06638433) and the other in the *ABCG1* gene on chromosome 21 (cg06500161)—were identified in the HyperGEN study in the basic (Fig. 1) and fully adjusted (Additional file 1: Figure S1) models. In the basic model adjusting for age, sex, cell count proportions, ancestry, and family relationship, MetS was associated with increased methylation at cg06500161 (*β* = 0.017, *p* value = 1.08 × 10^− 8^) and cg06638433 (*β* = 0.01, *p* value = 2.05 × 10^− 8^) (Table 2). After additionally adjusting for alcohol use and smoking, the associations remained consistent and statistically significant for cg06500161 (*β* = 0.02, *p* value = 2.60 × 10^− 8^) and closely approached statistical significance for cg06638433 (*β* = 0.01, *p* value = 3.10 × 10^− 7^). The results were consistent, but no longer statistically significant, in a sensitivity model additionally adjusted for left ventricular mass (cg06500161: *β* = 0.02, *p* value = 2.22 × 10^− 7^; cg06638433: *β* = 0.01, *p* value = 2.19 × 10^7^). The LVM adjusted results were similar after the FDR correction (adjusted *p* value = 0.054 for both cg06500161 and cg06638433). Mean DNA methylation level at each of the two CpG sites was significantly higher among HyperGEN participants meeting the criteria for an individual MetS component as well as overall for MetS status (Additional file 1: Figure S2).

**Table 2:** Top CpG methylation sites associated with metabolic syndrome status in HyperGEN discovery and REGARDS replication and meta-analysis in basic and adjusted models

Study	Model	CpG	Chr	Gene	Location	*β* (SE)	*p* value
Discovery set							
HyperGEN (*N* = 614 (basic) and 606 (fully adjusted))	Basic	cg06500161	21	*ABCG1*	43656587	0.017 (2.05 × 10^− 3^)	1.08 × 10^−8^
		cg06638433	17	*IGF2BP1*	47075175	0.01 (1.90 × 10^−3^)	2.05 × 10^−8^
	Fully adjusted	cg06500161	21	*ABCG1*	43656587	0.018 (3.20 × 10^−3^)	2.60 × 10^−8^
		cg06638433	17	*IGF2BP1*	47075175	0.01 (2.01 × 10^−3^)	3.10 × 10^−7^
Replication set							
REGARDS (*N* = 69)	Basic	cg06500161	21	*ABCG1*	43656587	0.029 (8.94 × 10^−3^)	0.001
		cg06638433	17	*IGF2BP1*	47075175	− 0.024 (0.012)	0.05
	Fully adjusted	cg06500161	21	*ABCG1*	43656587	0.032 (9.0 × 10^−3^)	4.7 × 10^−4^
		cg06638433	17	*IGF2BP1*	47075175	− 0.026 (0.012)	0.035
Meta-analysis							
HyperGEN and REGARDS	Basic	cg06500161	21	*ABCG1*	43656587	N/A	1.54 × 10^−10^
	Fully adjusted	cg06500161	21	*ABCG1*	43656587	N/A	2.15 × 10^− 10^
	Basic	cg06638433	17	*IGF2BP1*	47075175	N/A	1.29 × 10^−7^
	Full adjusted	cg06638433	17	*IGF2BP1*	47075175	N/A	1.55 × 10^−6^

Basic models refer to linear mixed models with metabolic syndrome as a predictor and CpG *β* score as the outcome, adjusted for age, sex, center, first four principal components, and five estimated cell proportions. Full adjusted models were additionally adjusted for smoking and alcohol use. Meta-analysis *p* values are from Han and Eskin [22] random effects models

Both cg06500161 and cg06638433 were examined for association with MetS in the REGARDS cohort. REGARDS participants with relevant data were overall 59.5 ± 4.5 years of age and 47.8% female (see Additional file 1: Table S2 for a description of the replication cohort by MetS status). Similar to HyperGEN, we observed increased DNA methylation levels at cg06500161 in both the basic (*β* = 0.029, *p* value = 0.001) and fully adjusted (*β* = 0.032, *p* value 4.7 × 10^− 4^) models in REGARDS. In meta-analysis of the combined HyperGEN and REGARDS cohorts, the association for cg06500161 remained consistent (meta-analysis for fully adjusted model: *p* value = 2.15 × 10^− 10^; Table 2). *IGF2BP1* cg06638433 was not associated with MetS in REGARDS, and the effect was in the opposite direction (Table 2).

**Fig. 2:**
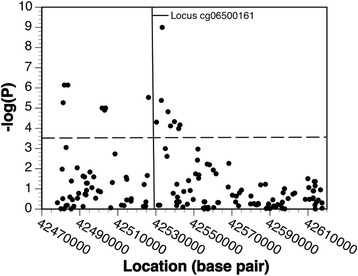
Associations between 154 *ABCG1* genetic variants within 20 kb (upstream and downstream) of the cg06500161 chromosome 21 locus and its methylation status (*cis*-meQTL). SNPs above the dashed line have *p* value for association > 0.05/154

**Fig. 3:**
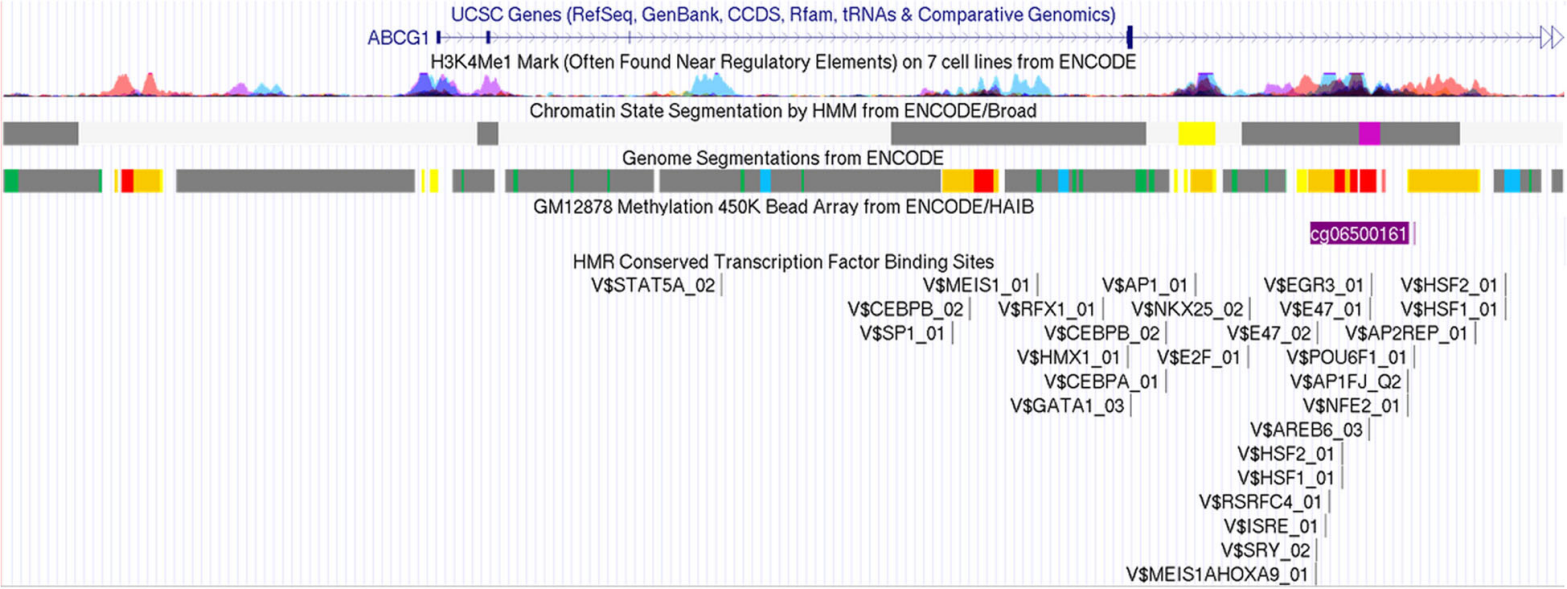
ENCODE plot with MetS signal of cg06500161. Genome segmentations from ENCODE color coding: bright red, predicted promoter region including transcription start site (TSS); light red, predicted promoter flanking region; orange, predicted enhancer; yellow, predicted weak enhancer or open chromatin *cis*-regulatory element; blue, CTCF (CCCTC-binding factor)-enriched element; dark green, predicted transcribed region; and gray, predicted low activity region

In the *cis*-meQTL analysis, 16 SNPs were significantly associated with methylation at the cg06500161 locus upon Bonferroni correction for multiple testing (3.2 × 10^− 4^ = 0.05/154 SNPs within 20 kb of cg06500161), reflecting potential contributions to the methylation signal from underlying common DNA sequence variants (Fig. 2). Among those 16 SNPs, 7 were in turn associated with MetS (*p* < 0.05) with the top SNP association signals being for rs17114493 and rs4547619 (Table 3). None of the 7 SNPs had previously been identified in GWAS of MetS or other metabolic traits. Those SNPs were associated with decreased methylation at cg06500161 yet increased MetS risk. In our primary analysis, MetS was associated with increased methylation at cg06500161. These results suggest these SNPs are not associated with MetS through this methylation site. Data from ENCODE shows that cg06500161 is a downstream of a predicted promoter region (Fig. 3).

**Table 3:** The results of *cis*-methylation quantitative trait loci for cg06500161 in *ABCG1* gene

CpG	SNP	Effect allele	*β* SNP-CpG	SE SNP-CpG	*p* value of SNP-CpG	OR SNP-MetS	SE SNP-MetS	*p* value of SNP-MetS
cg06500161	rs9978671	C	− 0.0203	0.0030	9.98 × 10^−10^	1.13	0.07	0.08
cg06500161	rs17114493	T	− 0.0264	0.0050	7.20 × 10^−7^	1.22	0.07	*4.30 × 10* ^*−3*^
cg06500161	rs4547619	C	− 0.0264	0.0050	7.20 × 10^−7^	1.22	0.07	*4.30 × 10* ^*−3*^
cg06500161	rs225440	C	0.0123	0.0025	2.96 × 10^−6^	0.91	0.07	0.19
cg06500161	rs225448	T	0.0123	0.0025	4.16 × 10^−6^	0.88	0.07	0.06
cg06500161	rs2839475	A	− 0.0220	0.0046	5.38 × 10^−6^	1.22	0.07	*5.94 × 10* ^*−3*^
cg06500161	rs1824010	C	−0.0222	0.0048	9.93 × 10^−6^	1.20	0.07	*8.98 × 10* ^*−3*^
cg06500161	rs8130198	A	− 0.0222	0.0048	9.93 × 10^−6^	1.20	0.07	*8.98 × 10* ^*−3*^
cg06500161	rs8134682	T	− 0.0222	0.0048	9.93 × 10^−6^	1.20	0.07	*8.98 × 10* ^*−3*^
cg06500161	rs8129752	A	− 0.0225	0.0049	1.27 × 10^−5^	1.21	0.07	*6.47 × 10* ^*−3*^
cg06500161	rs7283699	G	− 0.0131	0.0029	1.51 × 10^−5^	1.13	0.07	0.08
cg06500161	rs9974658	G	− 0.0131	0.0031	4.65 × 10^− 5^	1.11	0.07	0.17
cg06500161	rs915847	G	0.0118	0.0028	4.97 × 10^−5^	0.99	0.07	0.84
cg06500161	rs12329683	T	− 0.0130	0.0031	6.80 × 10^−5^	1.09	0.07	0.22
cg06500161	rs9982196	T	− 0.0120	0.0029	7.61 × 10^−5^	1.12	0.07	0.13
cg06500161	rs9978842	C	− 0.0144	0.0036	1.03 × 10^−4^	1.12	0.07	0.13

Linear mixed models were used for SNP-CpG association with CpG *β* score as the outcome adjusted for age, sex, study site, ancestry principal components, cell counts, and familial relationship. Logistic mixed models were used for SNP-MetS association with MetS as the outcome adjusted for age, sex, center, first four PCs, and familial relationship

*MetS* metabolic syndrome

## Discussion

In a sample of African-American adults with a 51% prevalence of MetS, we observed that MetS was associated with increased methylation on the cg06500161 locus in the *ABCG1* gene. This finding was successfully replicated in an independent sample of African-American adults in the REGARDS cohort, and meta-analysis of data from the two studies strengthened the association of the CpG with MetS. Methylation at cg06500161 (within a CpG island shore) [23] was associated with each MetS component suggesting this locus has pleiotropic effects. Evaluation of the region in ENCODE revealed the CpG is in a strong regulatory region which suggests that differential methylation at this locus could impact gene expression. An analysis of common SNPs in the region did not confirm SNPs influence on the relationship between the CpG and MetS.

This study is one of the firsts to report a potential epigenetic mechanism associated with a highly prevalent condition among African-American adults. Other studies have identified loci associated with MetS traits [24, 25], including central obesity, insulin responsiveness, and type 2 diabetes [26, 27], as well as lipid profiles in diverse populations [28, 29]. Das et al. identified decreased methylation at two loci of the *CPT1A* gene, involved in fatty acid oxidation, associated with MetS status in a Caucasian population, which corroborates findings by a prior study observing differential methylation at the same loci associated with triglycerides and very low-density lipoprotein cholesterol levels [25]. Methylation of the cg18181703 locus in the *SOCS3* gene, involved in leptin and insulin signaling, was also identified in a separate study, inversely correlated with BMI, triglycerides, and MetS but positively correlated with HDL-c levels [24]. Relevant to our findings, Yoon et al. independently identified *ABCG1* (cg06500161) as being associated with MetS (in a Caucasian Veteran population) using an Iterative Sure Independence Screening analysis approach [30]. *ABCG1* cg06500161 has also been reported to be associated with fasting insulin (in Caucasian non-diabetic adults) [26], blood lipids, and adiposity traits [29, 31]. Additionally, our results are consistent with a recent study of epigenetic determinants of type 2 diabetes in Mexican-Americans, showing differential DNA methylation levels at *ABCG1* [27]. Taken together, these studies suggest that epigenetic variations are strongly associated with MetS and its components and that *ABCG1* has been a consistent finding across diverse ethnic populations, strengthening the credibility of this gene’s relationship with metabolic traits.

*ABCG1* encodes for a protein included in the ATP-binding cassette (ABC) transporters superfamily, involved in the transport of molecules across extra- and intra-cellular membranes. *ABCG1* in particular plays an important role in macrophage cholesterol and phospholipid transport, and the regulation of cellular lipid homeostasis. Downregulation of *ABCG1* and *ABCA1* has been associated with reduced cholesterol efflux and has been shown to increase the risk of atherosclerosis [32]. Other studies have reported an association between *ABCG1* methylation and coronary artery disease [33, 34], and several studies report an association between liver X receptor, an inducer of ABC transporter gene expression, and cholesterol efflux which results in the inhibition of cell proliferation and stimulation of apoptosis in cancer [35, 36]. This evidence, taken together with the observation that alterations in the *ABCG1* gene were observed in 40% of invasive breast cancer tumors in the Cancer Genome Atlas [37, 38], provides a potential epigenetic link between MetS and chronic diseases including cancer that deserves further study. The higher methylation levels of *ABCG1* observed with MetS provide evidence for potential therapeutic targets that may be useful in drug development, and/or prevention of chronic diseases including CVD and cancer.

There are several limitations inherent in this study. First, HyperGen was a cross-sectional study of African-American adults, and this study design limits our ability to infer causality between MetS and DNA methylation. Prospective studies will be needed to definitively determine whether increased methylation of *ABCG1* is a consequence or cause of MetS. At least one Mendelian randomization study in Caucasian populations reported that lipids change methylation at cg06500161 suggesting the potential for a feedback loop mechanism that warrants further investigation [39]. In fact, if MetS affects methylation at *ABCG1*, this could help explain why our results did not suggest *cis*-meQTL SNPs influence the CpG-MetS relationship. Although we were able to identify significantly altered methylation on an *ABCG1* locus in relation to MetS among African-Americans that is consistent with epigenetic studies of metabolic traits in other ethnicities, improved understanding of racial differences in the biological mechanisms leading to significantly higher prevalence of MetS and components such as type 2 diabetes in African-Americans relative to other racial groups will require a larger study with adequate representation of multiple racial groups. DNA methylation was measured on stored and frozen buffy coat samples, which may have been altered during the freezing and thawing process; however, buffy coat samples are widely used for methylation analysis due to the high concentration of DNA. A major strength of the study is the use of epigenome-wide methylation analysis using a wide panel of CpG markers on understudied African-Americans and replication in a separate independent sample of African-American participants.

## Conclusion

In conclusion, MetS in African-American adults was associated with increased methylation at the cg06500161 locus in the *ABCG1* gene, located in a highly conserved regulatory region of the genome. *ABCG1*, involved in cholesterol and phospholipid transport, has been associated with MetS and component traits in other studies. The high prevalence of MetS among African-Americans and the consistent associations between MetS and many chronic diseases such as cancer highlight the need for future prospective confirmatory studies that may inform clinical strategies and interventions.

## Additional file

Additional file 1: **Table S1.** HyperGEN family structure. **Table S2.** Baseline characteristics of REGARDS study participants. **Figure S1.** Epigenome-wide association for metabolic syndrome among African-Americans in HyperGEN study (*N* = 606). Figure S2. Differences in mean DNA methylation (%) of (A) cg06500161 in ABCG1 and of (B) cg06638433 in IGF2BP1 gene by metabolic syndrome and components. (PDF 1194 kb)
