# Supplementary material for: Epigenome-wide association study of metabolic syndrome in African-American adults
Source: Clin Epigenetics. 2018 Apr 10;10:49. doi: 10.1186/s13148-018-0483-2 (PMC5891946; doi:10.1186/s13148-018-0483-2)
Supplement: Supplementary file 1 — Table S1. HyperGEN family structure. Table S2. Baseline characteristics of REGARDS study participants. Figure S1. Epigenome-wide association for metabolic syndrome among African-Americans in HyperGEN study (N = 606). Figure S2. Differences in mean DNA methylation (%) of (A) cg06500161 in ABCG1 and of (B) cg06638433 in IGF2BP1 gene by metabolic syndrome and components. (PDF 1194 kb) [file 13148_2018_483_MOESM1_ESM.pdf]

Additional file 1

Epigenome-wide Association Study of Metabolic Syndrome in African-American Adults

Tomi Akinyemiju, Anh N. Do, Amit Patki, Stella Aslibekyan, Degui Zhi, Bertha Hidalgo, Hemant K Tiwari, Devin Absher, Xin Geng, Donna K Arnett, Marguerite R. Irvin

Table S1. HyperGEN family structure

	**Family size**				
	**1**	**2**	**3**	**4**	**5**
Number of members per family	334	82	30	4	2
Number of parent-child pairs	46				
Number of full sibling pairs	42				

Table S2. Baseline characteristics of REGARDS study participants.

	**MetS+**	**MetS-**	**P-value**[a]
N	37	32	
Age[b]	59.7± 4.8	59.3 ± 4.2	0.74
High WC/ Obesity			
WC (cm)[b]	105.3 ± 17.8	87.2 ± 11.4	<0.0001
BMI[a]	31.8 ± 5.1	26.7 ± 4.0	<0.0001
Elevated Triglycerides			
Triglycerides (mg/dL)[c]	131.8 ± 75.6	94.9 ± 80.2	0.02
Reduced HDL Cholesterol			
HDL-Cholesterol (mg/dL)[b]	45.8 ± 13.8	59.4 ± 20.5	0.002
Elevated Blood Pressure			
DBP (mmHg)[b]	80.1 ± 12.5	73.0 ± 10.9	0.01
SBP (mmHg)[b]	141.6 ± 22.8	117.6 ± 18.2	<0.0001
Hypertension (%)	81.1	12.5	<0.0001
Elevated Fasting Glucose			
Fasting glucose (mg/dL)[b]	119.7 ± 32.8	97.3 ± 30.7	0.005
Metabolic components (%)[d]			
0 components (%)	0	40.6	
1 components (%)	0	31.3	
2 components (%)	0	28.1	
3 components (%)	29.7	0	
4 components (%)	35.1	0	
5 components (%)	35.1	0	

[a]Significance determined using Chi-square test for categorical, t-test for continuous, or kruskal.test test for non-parametric continuous variables.
[b]Presented as mean (standard deviation) for normal continuous characteristics.
[c]Presented as median (interquartile range) for non-parametric continuous characteristics.
[d]Metabolic components are high waist circumference (WC), elevated triglycerides, reduced HDL cholesterol, elevated blood pressure, and elevated fasting glucose.
BMI, body mass index; DBP, diastolic blood pressure; HDL, high-density lipoprotein; LDL, low-density lipoprotein; SBP, systolic blood pressure; WC, waist circumference

Figure S1. Epigenome-wide association for metabolic syndrome among African-Americans in HyperGEN study (N=606).

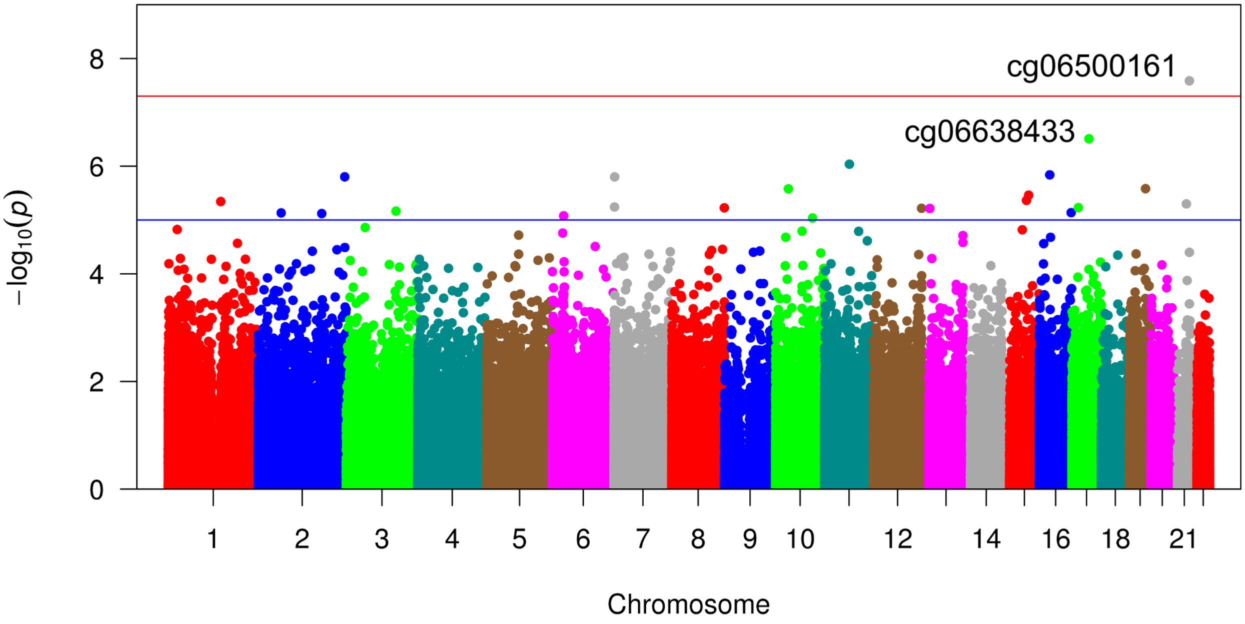

Fully adjusted model includes age, sex, center, first four principal components, five estimated cell proportions, batch effect, family structure, smoking, and alcohol

Figure S2. Differences in mean DNA methylation (%) of (A) cg06500161 in ABCG1 and of (B) cg06638433 in IGF2BP1 gene by metabolic syndrome and components.

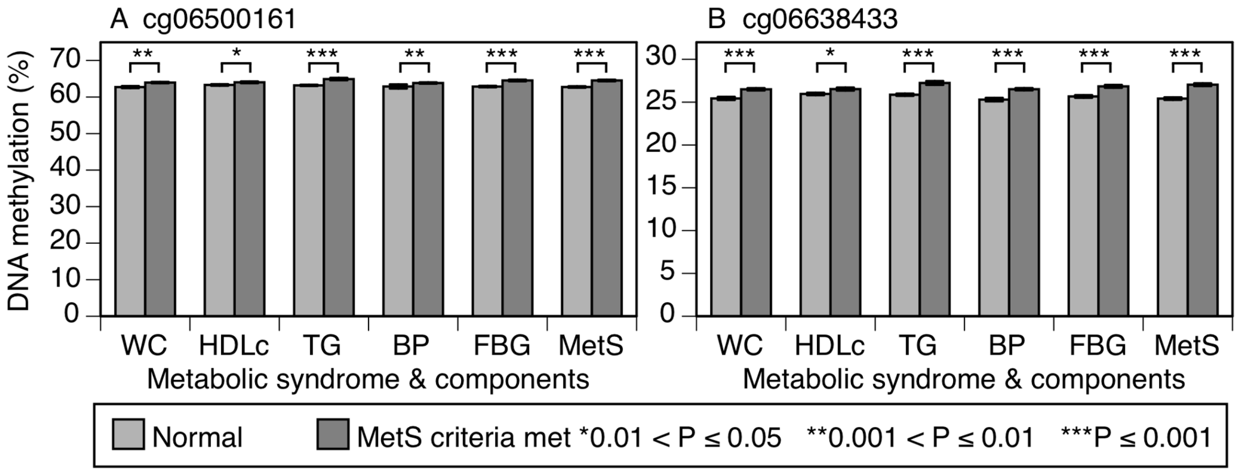

X-axis shows the criteria met (yes/no) for each component of MetS and MetS; Y-axis the mean DNA methylation (%) with error bars showing standard error. MetS criteria met: WC–waist circumference ≥102 cm for men and ≥88 cm for women, HDLc –high density lipoprotein cholesterol <40 mg/dL for men and <50 mg/dL for women, TG–triglycerides ≥150 mg/dL, BP–blood pressure ≥130/≥85 mm Hg, and FBG–fasting blood glucose ≥100 mg/dL.
